# Long-Term Effect of Daily Chemical Disinfection on Surface Topography and *Candida Albicans* Biofilm Formation on Denture Base and Reline Acrylic Resins

**DOI:** 10.3290/j.ohpd.a45521

**Published:** 2020-11-20

**Authors:** Maria Isabel Amaya Arbeláez, Carlos Eduardo Vergani, Paula Aboud Barbugli, Ana Claudia Pavarina, Paula Volpato Sanitá, Janaina Habib Jorge

**Affiliations:** a Postgraduate student, São Paulo State University (UNESP), Araraquara Dental School, Campus Araraquara, SP, Brazil. Acquisition of data; analysis and interpretation of data; critical revision of the article and final approval of the version to be published.; b Professor, São Paulo State University (UNESP), Araraquara Dental School, Campus Araraquara, SP, Brazil. Participation in the interpretation of results this study.; c Professor, São Paulo State University (UNESP), Araraquara Dental School, Campus Araraquara, SP, Brazil. Acquisition of data; participation in the interpretation of results this study; critical revision of the article and final approval of the version to be published.; d Professor, São Paulo State University (UNESP), Araraquara Dental School, Campus Araraquara, SP, Brazil. Participation in the interpretation of results this study.; e Professor, São Paulo State University (UNESP), Araraquara Dental School, Campus Araraquara, SP, Brazil. Critical revision of the article and final approval of the version to be published.; f Professor, São Paulo State University (UNESP), Araraquara Dental School, Campus Araraquara, SP, Brazil. Conception and design of the study; responsible for the organisation of the literature review necessary for the experimental design phase and discussion of manuscript and references; participation in the interpretation of results this study; critical revision of the article and final approval of the version to be published.

**Keywords:** biofilm, acrylic base resin, hard relines, roughness, denture cleansers

## Abstract

**Purpose:**

This study investigated the effect of long-term daily chemical disinfection on the topographic and *Candida albicans* biofilm formation on a denture base resin and a reline acrylic resin.

**Material and Methods: Circular samples (14 × 1.2 mm) were fabricated from a denture base (Vipi Wave) and reline acrylic resins (Tokuyama Rebase Fast II). Samples were kept in 50 ml of distilled water (48 h at 37°C). Subsequently, the samples were immersed in five different solutions:**

0.5% sodium hypochlorite; 3.8% sodium perborate; 2% chlorhexidine gluconate; apple vinegar containing 4% maleic acid; and distilled water (control group). The specimen was immersed in the solutions for 8 h daily and transferred to distilled water at 37°C for more 16 h. The surface topographic and *Candida albicans* (ATCC 90028) biofilm formation were evaluated at baseline (before chemical disinfection) and after 1, 3 and 6 months of immersion. The surface topographic was evaluated by arithmetical roughness average (Ra) and scanning electron microscope (SEM), while the biofilm formation was evaluated by colony-forming units (CFU/ml) method and Alamar Blue assay (cell metabolism). The results were evaluated by three-way analysis of variance (ANOVAs) and post-hoc tests (α = 0.05).

**Results:**

The results showed statistically significant effects from the type of acrylic resin (p = 0.029) and time (p <0.001) on the roughness of the specimen. In general, the reline resin had higher roughness than the denture base resin. In addition, the roughness of the samples after 1, 3 and 6 months of immersion in the cleaning solutions was higher than at baseline. In relation to the microbiological assays, there were no statistically significant differences (p >0.055) in the CFU/ml values of the biofilms among the different resins, periods of time and cleaning solutions. Considering the metabolism of the cells within the biofilms, the results showed that, at baseline, it was statistically significantly higher (p <0.05) than after 1, 3 and 6 months of storage. The SEM images showed that all disinfectant solutions provided surface changes of both acrylic resins (base and reline) after 1, 3 and 6 months of immersion.

**Conclusions:**

The roughness of both acrylic resins was affected by the disinfection in all cleaning agents, increasing over time, and this effect was more evident in the reline acrylic resin group. This surface change was also observed in the SEM images. While the number of cells within the biofilms was not affected by immersion in the cleaning agents, their metabolism was lower after 1, 3 and 6 months of immersion.

The removable dentures are made from acrylic resins, which are classified by the polymerisation method as autopolymerised, heat-polymerised, visible light polymerised and microwave polymerised.^1^ The reline materials are autopolymerised acrylic resins specially formulated for direct rebasing of dentures. Since the reline materials are in direct contact with the mucosa, its mechanical, physical, and biological properties should be similar to those of heat-polymerised resins used to fabricate denture bases. However, the variations in chemical composition and purity of commercial resin systems, the degree of conversion of the monomers, and the manipulation variables may influence the biological, physical, and mechanical properties of denture base and reline acrylic resins. This fact may result in a relevant clinical impact to denture users, since it can increase the acrylic surface irregularity, creating a reservoir of microorganism.^7^ It is also important to mention that the microorganisms can penetrate into these irregularities and survive to a depth ranging 1.0 to 2.0 μm.^8^ This aspect can facilitate the onset and development of diseases, such as denture stomatitis.

Denture stomatitis is an erythematous inflammatory lesion of the oral mucosa, which occurs in dentures wearers. The contamination by *Candida* spp. seems to be the initial factor for the start and spread of the disease.^12^ These microorganisms have the ability to colonise and adhere to both, oral tissues and acrylic resins, and they are able to congregate with bacteria of the oral cavity, establishing a complex structure covered by extracellular matrix, named biofilm.^4^ Biofilms are resistant to many types of drugs and are able to tolerate high concentrations of antifungal agents.^42^ For this aspect, the treatment of denture stomatitis has been a challenge in clinical practice due to its high prevalence and the high number recurrence after treatment with antifungals.^21^

Different techniques of dentures disinfection have been proposed in order to prevent the onset and recurrence of denture stomatitis. The disinfection protocol that combines mechanical cleaning (brushing) and soaking in disinfectant solutions (chemical disinfection) has been indicated because it reduces the formation of microbial biofilms on dentures.^43^,^52^ However, the abrasive action of this disinfection protocol can change the roughness of denture material, causing microorganism growth and biofilm accumulation.^54^ Furthermore, elderly persons with physical disabilities, whose manual dexterity is often limited, may prefer an alternative method to cleaning dentures, which is the use of disinfectant solutions.^3^ There are several solutions with antimicrobial properties that can be used for dentures disinfection. Chlorhexidine is currently considered the standard disinfectant solution and it is the substance most extensively studied in the dental field because of its antimicrobial properties.^5^,^7^ Against *C. albicans* biofilm, it is effective associated with mechanical brushing or as a cleaning agent.^38^ Sodium hypochlorite also has antifungal activity against *Candida* species with short periods of immersion.^5^,^11^,^52^ Sodium perborate has an effervescent function that promotes mechanical removal of debris by the release of oxygen during its reaction with water. As disinfectant solution, it reduced the amount of *C. albicans* after 60 min soaking time.^5^,^7^,^19^ The apple vinegar is another solution with fungicidal activity against *Candida albicans* after 120 min of immersion at concentrations of 10 mg/ml.^32^

It is important to emphasise that these disinfectant solutions should be effective against microorganisms without causing harmful effects on the physical and mechanical properties of the materials used for base or relining of dentures. The effects of soaking on chlorhexidine on the physical and mechanical properties of denture base and reline acrylic resins are still contradictory. It has been demonstrated that soaking for 10–60 min in 4% chlorhexidine resulted in changes on surface roughness of the materials,^7^,^39^ although other authors did not corroborate these findings.^2^,^29^ Sodium hypochlorite and sodium perborate may also have harmful effects on roughness of the acrylic materials. Because of the corrosive power of sodium hypochlorite, the surface of the acrylic resin may become more porous.^17^,^34^ In fact, Paranhos et al^37^ and Fernandes et al^13^ observed that immersion in sodium hypochlorite solution caused increase in the surface roughness of an acrylic resin when the disinfection simulated an overnight use for 1 year and a half^37^ or was done during 30 min.^13^ Sodium perborate is highly alkaline and has high level of oxygen. This facilitates the dissolution of resin matrix components or cause absorption of water or saliva,^17^ resulting in an increase in surface roughness.^7^,^28^ Mota et al^32^ observed that the apple vinegar was capable of preventing the adhesion of microorganisms on acrylic resins samples without causing color change and surface roughness after immersion in the solution for 2 h, despite there are few studies that investigated this solution.

All studies mentioned earlier evaluated the effect of the disinfectant solutions on roughness or other physical properties of the acrylic resins without concern about its influence on biofilm formation. To our knowledge, there are only two studies that used the same disinfection protocol to evaluate the physical and mechanical properties, as well as the biofilm formation on the acrylic resin after immersion and brushing with disinfectant solutions.^35^,^36^ However, the immersion time evaluated by them was 10 s. Evaluating a longer period of immersion is important because the cumulative effect of the solutions can cause irreversible changes in the structure of polymer, increasing the roughness of the material and promoting the accumulation microorganisms. In addition, the currently disinfection protocol adopted for denture disinfection is daily, overnight. Therefore, the purpose of this study was to investigate the effect of long-term daily chemical disinfection on the topographic and *C. albicans* biofilm formation on a denture base resin and a reline acrylic resin. The null hypothesis was that the immersion in disinfectant solutions would not influence the topography and biofilm formation on the samples.

## Material And Method

### Samples Fabrication

Denture base and reline acrylic resins (Table 1) were selected for this study because, owing to differences in their chemical composition and water sorption, they are differently affected by water immersion. Circular samples of denture base acrylic (N = 320) resin were made in stainless steel moulds (14 mm × 1.2 mm) that were placed in dental flasks, sandwiched between two glass plates. The glass was blasted with aluminium oxide to create a 3.0 μm of surface roughness, simulating the roughness found on the inner surface of the denture.^20^ The acrylic resin was portioned according to the manufacturer’s instructions. The matrix was filled with the acrylic resin (plastic phase) and the flask was pressured in a hydraulic press with 1.0 kg/f, and gradually increased to 1.5 kg/f. Then, the samples were polymerised according to manufacturer’s instructions. To prepare the circular samples of Tokuyama Rebase Fast II (N = 320), a metal matrix (14 mm × 1.2 mm in thickness) was fixed to sprayed glass plate with aluminium oxide (3.0 μm surface roughness). The reline resin was manipulated according to the manufacturer’s instructions and inserted into the matrix breakaway compartment. After insertion, another sprayed glass plate was placed over the matrix, and light pressure was applied to expel excess material from the mould.^15^ The reline resin samples were produced at room temperature (25°C), according to the instructions of the manufacturer. The boundaries of the samples were trimmed out with a maxi cut bur (Lesfils de August Malleifer). A total of 320 samples of each acrylic resin were fabricated. In order to remove the residues, all samples were washed in an ultrasonic bath using distilled water throughout 15 min.

**Table 1:** Denture base acrylic resins tested

Material	Composition		Powder/Liquid ratio	Polymerization cycle	Lot number
	Powder	Liquid			
Tokuyama Rebase Fast II	PEMA	AAEM and 1,9-nonanediol dimethacrylate	2.1 g/1.0 ml	5.5 min at room temperature	225EZ3
Vipi Wave	PMMA, benzoyl peroxide, pigments	MMA, EGDMA, inhibitor	2.15 g/1.0 ml	10 min at 20% power, followed by 4 min at 60% power	75643

PMMA, poly(methyl methacrylate); MMA, methyl methacrylate; EDGMA, ethylene glycol dimethacrylate; PEMA, poly(ethyl methacrylate); AAEM, 2-acetoacetoxy (ethyl) methacrylate.

### Roughness

The surface roughness of the specimen was measured according to Izumida et al,^20^ using the same methodology. Samples were measured with a surface roughness profilometer (SJ 400; Mitutoyo, Kawasaki, Japan). The resolution was 0.01 μm, interval (cut-off length) 0.8 mm, transverse length 2.4 mm and stylus speed 0.5 mm/s. In the central area of each specimen, three measurements were made, and the average reading was designated as the intact Ra value for that specimen.^35^ Only samples with roughness values between 2.5 µm and 3.5 µm were selected. Then, the denture base and reline acrylic resin samples were stored in 50 ml of distilled water for 48 h at 37°C in order to eliminate residual monomer.^25^

### Experimental Groups

First, both sides of each sample were disinfected using ultraviolet light under dry conditions for 20 min.22–25 Then, they were randomly distributed into five groups according to the cleansing agent tested: 0.5% sodium hypochlorite (Farmácia Reativa, Araraquara, SP, Brazil), 3.8% sodium perborate (Farmácia Reativa, Araraquara, SP, Brazil), 2% chlorhexidine gluconate (Farmácia Reativa, Araraquara, SP, Brazil), Minhoto apple vinegar pure containing 4% maleic acid, and distilled water (control group). The specimen was immersed in the solutions for 8 h daily and transferred to distilled water at 37°C for more 16 h, in order to simulate overnight disinfection and daily denture use, respectively.^37^ According to Shay,^44^ some cleansers were more effective when used overnight.

**Fig 1:**
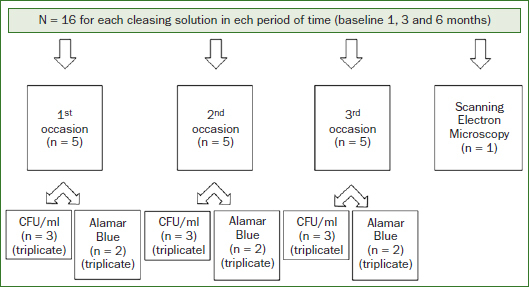
Diagram of the experiment (N = 320).

Topographic and *Candida albicans* biofilm formation on denture base and reline acrylic resins specimen were evaluated at baseline (before chemical disinfection) and after 1, 3 and 6 months of immersion. The surface topographic was evaluated by Ra (n = 16) and scanning electron microscope (SEM) (n = 1), while the biofilm formation was evaluated CFU/ml method (n = 9) and Alamar Blue assay (n = 6). All experiments were performed three independent occasions. In addition, at each time-point 16 samples were destroyed and were not available for further experiments. The diagram of all experimental conditions is illustrated in Figure 1.

### Surface Topography Assessments

The surface topographic of the acrylic samples was evaluated by roughness parameters and SEM. The surface roughness of the specimen was measured as described above at baseline (before chemical disinfection) and after 1, 3 and 6 months of immersion, always before the onset of the microbiological experiments. For SEM analysis, a specimen from each group was metallised with gold layer and positioned in the scanning electron microscope (JEOL JSM-6610LV), to obtain four pictures. The final magnifications for each view were 50×, 100×.

### Microbiological Assays

*Candida albicans* (*C. albicans*) strain (American Type Culture Collection ATCC 90028) was used in the microbiological assays. To prepare the inoculum, a loopful of the stock culture was streaked onto Sabouraud Dextrose Agar (SDA) with chloramphenicol and incubated at 37°C for 48 h. Five colonies of the culture were transferred to 10 ml of yeast nitrogen base (YNB) culture medium supplemented with 50 mM glucose and incubated at 37°C for 16 h. Then, a dilution of 1:20 of the yeast culture was incubated for more than 8 h. After incubation, the cells were centrifuged twice and washed with sterile phosphate-buffered saline (PBS) (pH 6.8) at 5000 rpm for 10 min, and resuspended in RPMI-1640 culture medium (Sigma, St Louis, MO) to achieve hyphae form, a crucial step in the initiation of candidiasis. The optical density of the suspension was measured and standardised to a concentration of 1 × 10^6^ cells/ml.

The biofilms were grown on 24-well, flat-bottom microtiter plate (TPP TechnoPlastic Products) containing the samples. Aliquots of 1500 μl of the inoculum and RPMI-1640 culture medium was added to each well and then incubated at 37°C (75 rpm) in an orbital shaker for 90 min (adhesion phase). The non-adherent cells were removed from the specimen by carefully washing twice with PBS buffer and 1500 μl of the culture medium was placed in each well and maintained for 24 h at 37°C in an orbital shaker (75 rpm). After 24 h, 750 μl of RPMI-1640 medium was removed and an equal volume of fresh medium was added. The 24-well microplates containing the samples were then incubated for more 24 h. After the 48 h of biofilm formation, the culture medium was aspirated and the non-adherent cells were removed by washing twice with PBS buffer. The biofilms were evaluated using the colony count method (CFU/ml) and the Alamar Blue to assess the cells metabolic activity at baseline (before chemical disinfection) and after 1, 3 and 6 months of immersion.

### Colony Count Method (CFU/ml)

The samples were transferred to new 24-well microplates and the bioﬁlms were disrupted and removed scraping the disks’ surface thoroughly with a scraper for 3 minutes^51^ and serial tenfold dilutions were performed. Then, an aliquot of 25 μl of the 10-3 and 10-4 dilutions were transferred to Petri dishes containing SDA and plates were incubated at 37°C for 48 h. After this period, the plates were placed on a colony counter and the number of colonies was determined and calculated (CFU/ml).

### Alamar Blue Assay

The Alamar Blue assay measures the cell metabolism based on enzymatic reduction of indicator dye by the viable cells. Immediately after the adhesion phase and removal of the non-adherent cells, 1500 μl of RPMI-1640 culture medium and 150 μl of Alamar Blue solution (Thermo Fisher Scientific) were added on each well. The 24-well plates were incubated at 37°C for 48 h in an orbital shaker (75 rpm). Then, aliquots of 200 µl of the final solution were transferred to wells of a black 96-well microtiter plate and the fluorescence of the samples was measured using Fluoroskan Ascent FL (Lab Systems) with a filter of 544 nm emission and 590 nm transmission.

### pH Values

To determine the acidity of the solutions used in this study, the pH values were measured using a digital pH meter (Quimis, Model Q400AS). The acidic pH can promote the degradation of the acrylic resin surface and, hence, influence the formation of biofilms.

### Statistical Analysis

Initially, the CFU ml−1 values were transformed into log_10_ and the homogeneity of variance and normality of the data were verified by the Levene and Shapiro–Wilk tests, respectively. The homogeneity of variance and normality of the data was confirmed. The three factors considered in the present investigation were ‘type of acrylic resin’ (denture base resin and reline acrylic resin), ‘cleaning agent’ (distilled water, sodium perborate, chlorhexidine, sodium hypochlorite, and vinegar) and ‘period of time’ (baseline, 1, 3 and 6 months). To assess if these factors affected the roughness of the two acrylic resins, the results were submitted to a three-way ANOVA followed by Bonferroni post-hoc test. To evaluate the results from microbiological assays, the log_10_ CFU/ml and fluorescence values (Alamar Blue assay) were analysed by a three-way ANOVA followed by Tukey post-hoc test. A statistical significance level of 95% was adopted for all tests. Data were presented as arithmetic mean ± standard deviations (SD).

## Results

**Fig 2:**
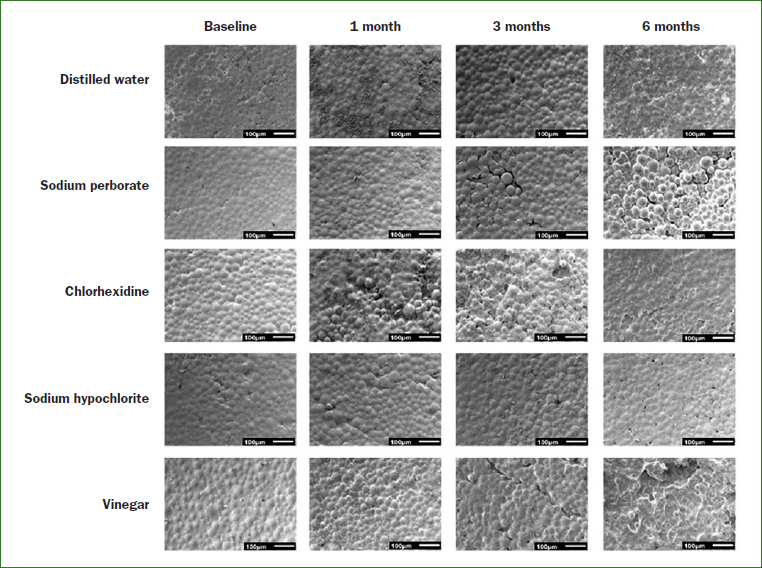
Mean values and standard deviation of roughness of all groups. Different letters represent statistically significant differences for the effect ‘period of time’ (Bonferroni post-hoc test at p <0.05).

The three-way ANOVA found statistically significant effects of the factors ‘type of acrylic resin’ (p = 0.029) and ‘period of time’ (p <0.001) on the roughness of the samples (Table 2). Within each immersion period, the different cleaning agents used caused no statistically significant effect (p = 0.918) on the roughness of both acrylic resins’ samples. When the factor ‘type of acrylic resin’ was considered (Fig 2), the results showed that the mean values of roughness of the reline acrylic resin samples were higher (4.09 μm) than those from the denture base acrylic resin samples (4.02 μm). When the factor ‘period of time’ was evaluated (Fig 2), it was observed that there was a statistically significant increase in the roughness of the samples after immersion in the cleaning agents in all periods when compared to baseline (mean value = 3.10 μm). The long-term evaluation showed that after 1 month of immersion in the cleaning agents the roughness of the samples was similar to that observed after 6 months of daily disinfection (mean values = 4.53 μm and 4.56 μm, respectively), being both statistically significantly higher (p <0.05) than that observed after 3 months of immersion in the cleaning agents (mean value = 3.95 μm).

**Table 2:** Three-way ANOVA values for specimen surface roughness

Effect	SS*	df	MS	F	p
**Between groups**					
Acrylic resin	0.818	1	0.818	4.839	0.029*
Disinfectant solution	0.159	4	0.040	0.235	0.918
Acrylic resin × disinfectant solution	0.496	4	0.124	0.733	0.571
Residue	23.669	140	0.169		
**Within groups**					
Period of time	223.934	3	74.645	522.679	0.000*
Period of time × acrylic resin	0.500	3	0.167	1.167	0.322
Period of time × disinfectant solution	1.732	12	0.144	1.010	0.438
Period of time × acrylic resin × disinfectant	0.418	12	0.035	0.244	0.996
residue	59.981	420	0.143		

df: Degrees of freedom; *statistically significant at the 5% level.

It was observed that none of the factors considered had a statistically significant effect on the log_10_ CFU/ml values of the *C. albicans* biofilm (Table 3). The mean values of log_10_ CFU/ml and SDs obtained from each experimental group after biofilm formation are shown on Table 4. As can be seen, *C. albicans* formed biofilm on both types of resin after disinfection for all periods of time. The other microbiological parameter evaluated was the cell metabolism of the biofilms measured by the Alamar Blue fluorescence values. The results showed that only the factor ‘period of time’ had a statistically significant effect (p <0.001) on this parameter (Table 5). As can be seen on Table 6, the fluorescence values obtained at baseline (time 0) for the biofilms of all experimental groups was statistically significantly higher (p <0.05) than the fluorescence values found at 1, 3 and 6 months of chemical disinfection. There were no statistically significant differences in the fluorescence values obtained after 1, 3 and 6 months of immersion (p >0.05).

**Table 3:** Summary of three-way ANOVA for the log_10_ CFU/ml values of the *C. albicans* biofilm

Effect	SS	df	MS	F	p
Acrylic resin	0.70	1	0.697	4.6000	0.055
Period of time	0.20	3	0.065	0.4300	0.732
Cleansing agent	0.49	4	0.122	0.8100	0.524
Acrylic resin × period of time	0.23	3	0.076	0.5000	0.682
Acrylic resin × cleansing agent	0.26	4	0.066	0.4400	0.783
Period of time × cleansing agent	1.17	12	0.097	0.6400	0.800
Period of time × acrylic resin × cleansing agent	0.84	12	0.070	0.4600	0.931
Residue	11.66	77	0.151		

df: Degrees of freedom.

**Table 4:** Mean values of log_10_ CFU/ml and standard deviations (SD) from *C. albicans* biofilms of all experimental conditions

Resin	Disinfectant solution	Storage time (months)			
		0	1	3	6
Denture base	Water	6.84 (0.58)	6.65 (0.02)	6.70 (0.60)	7.05 (0.42)
	Perborate	6.92 (0.08)	6.73 (0.08)	6.70 (0.38)	6.69 (0.58)
	Chlorhexidine	6.46 (0.53)	6.77 (0.05)	6.64 (0.59)	6.55 (0.06)
	Hypochlorite	6.83 (0.42)	6.45 (0.09)	6.68 (0.61)	6.43 (0.09)
	Vinegar	6.66 (0.57)	6.60 (0.05)	6.89 (0.37)	6.93 (0.48)
Reline resin	Water	6.95 (0.22)	7.17 (0.54)	6.36 (0.29)	7.12 (0.51)
	Perborate	6.96 (0.22)	6.87 (0.49)	6.87 (0.06)	6.69 (0.64)
	Chlorhexidine	7.04 (0.27)	6.95 (0.33)	6.62 (0.14)	6.96 (0.19)
	Hypochlorite	6.82 (0.04)	6.77 (0.10)	7.02 (0.54)	6.64 (0.08)
	Vinegar	7.01 (0.08)	6.73 (0.29)	6.54 (0.09)	6.83 (0.09)

**Table 5:** Three-way ANOVA fluorescence values of C. albicans biofilm

Effect	SS	df	MS	F	p
Acrylic resin	162,683.0	1	162,683.0	2.57	0.113
Period of time	3,256,890.0	3	1,085,630.0	17.17	<0.001*
Cleansing agent	74,056.0	4	18,514.0	0.29	0.882
Acrylic resin × period of time	29,537.0	3	9846.0	0.16	0.926
Acrylic resin × cleansing agent	64,866.0	4	16,216.0	0.26	0.905
Period of time × cleansing agent	471,015.0	12	39,251.0	0.62	0.819
Period of time × acrylic resin × cleansing agent	108,378.0	12	9031.0	0.14	1.000
Residue	4,996,394.0	79	63,245.0		

df: Degrees of freedom; *statistically significant at the 5% level.

**Table 6:** Mean fluorescence values of Alamar Blue and standard deviations (SD) from C. albicans biofilms of all experimental conditions

Resin	Disinfectant solution	Storage time (months)			
		0	1	3	6
Denture base	Water	2291 (137)*	1735 (231)	1639 (352)	2067 (291)
	Perborate	2215 (157)*	1784 (77)	1861 (412)	1986 (218)
	Chlorhexidine	2289 (144)*	1869 (233)	1846 (516)	2012 (483)
	Hypochlorite	2323 (130)*	2055 (114)	1894 (371)	1973 (366)
	Vinegar	2263 (104)*	1910 (178)	1898 (442)	1781 (300)
Reline resin	Water	2361 (62)*	1919 (104)	1894 (294)	2076 (226)
	Perborate	2328 (27)*	2015 (242)	1870 (314)	2095 (298)
	Chlorhexidine	2278 (61)*	1848 (251)	1983 (180)	2004 (232)
	Hypochlorite	2303 (99)*	2021 (135)	1953 (174)	2031 (289)
	Vinegar	2350 (104)*	1968 (127)	2045 (188)	1829 (152)

*Statistically significant differences from baseline (time 0) in relation to all periods of time – horizontally (Tukey post-hoc test at p <0.05).

**Fig 3:**
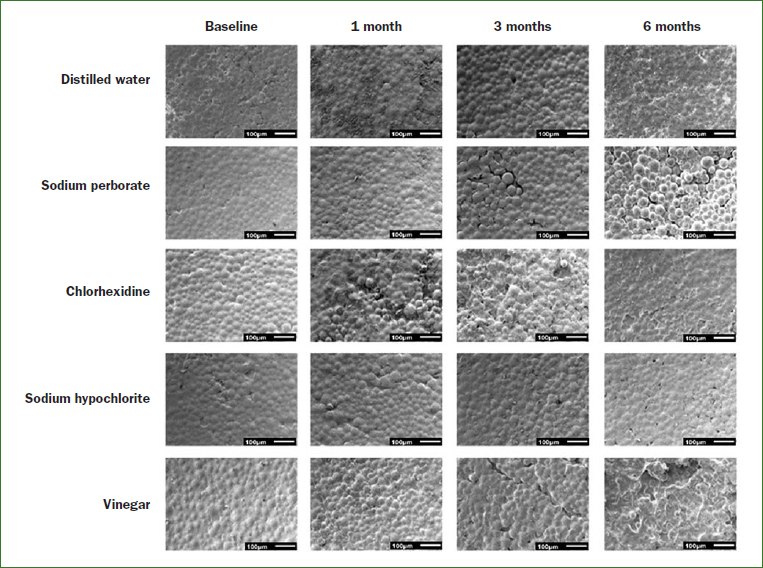
Topographic images obtained from the SEM (magnification 100×) illustrating the changes of the polymer matrix of the denture base acrylic resin in the different immersion times.

**Fig 4:**
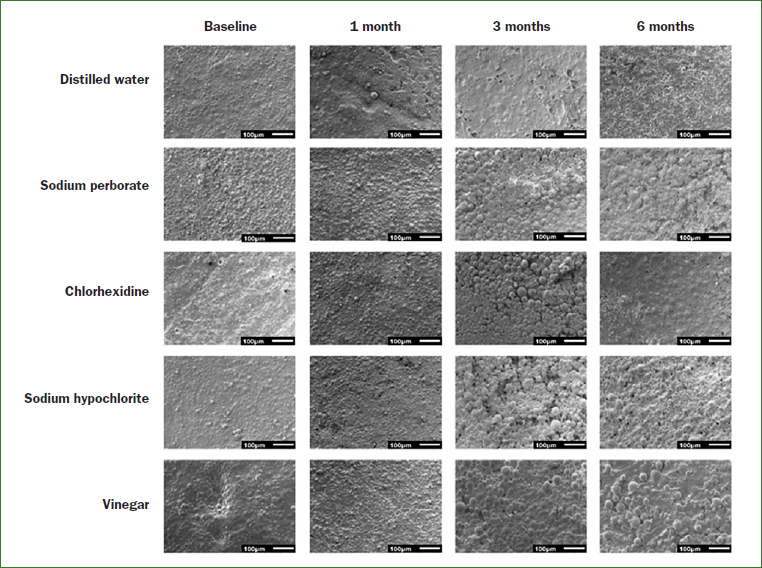
Topographic images obtained from the SEM (magnification 100×) illustrating the changes of the polymer matrix of the reline acrylic resin in the different immersion times.

From the analysis of the images obtained by SEM (Figs 3 and 4), it was possible to observe that immersion in all cleaning solutions caused a degradation of the samples surface over time. It was also noted that immersion of the samples in apple vinegar resulted in major changes in the acrylic surface (Figs 3 and 4).

In terms of the pH of the solutions used in this study, the following values were found: Distilled water: 7.2; Chlorhexidine digluconate: 6.7; Sodium perborate: 3.9; Sodium hypochlorite: 5.1; Vinegar: 2.8. The pH values of all cleaning agents used in the present study are described in Table 7.

**Table 7:** pH values of the disinfection solutions tested

Disinfectant solutions	ph
Distilled water	7.2
Chlorhexidine digluconate	6.7
Sodium perborate	3.9
Sodium hypochlorite	5.1
Vinegar	2.8

## Discussion

Some characteristics of the acrylic resins of the dentures, as roughness, hydrophobicity, and electrostatic forces may promote and/or facilitate the adhesion of microorganisms, resulting in biofilm formation.^27^ The effective removal of this biofilm is essential not only to the maintenance of the oral health, but also to prevent and treat the oral infections. There are several techniques and chemical solutions available to cleaning dentures. Regardless of which protocol is used, the routine use of denture cleansers can adversely affect the physical, chemical and/or biological properties of the denture base and reline acrylic resins, resulting in surface changes that may promote the adhesion of microorganisms, biofilm formation, and, consequently, appearance of fungal infections.^47^,^53^ Thus, the current study investigated if immersion of two types of acrylic resins in different solutions for long periods of time could alter the topography of the resins and the ability of *Candida albicans* to form biofilms.

Chemicals solutions, such as 0.5% sodium hypochlorite, 2% chlorhexidine, 3.8% sodium perborate and pure apple vinegar have been widely used because of their antiseptic properties and ability to reduce the amount of biofilm.^5^,^11^ These cleaning agents should be effective without causing harmful effects on the physical and mechanical properties of the denture reline materials and denture base acrylic resins. Changes in the surface morphology of denture base acrylic resins have been detected after exposure to some denture cleansers.^7^,^28^ In the present study, the type of cleaning agent had no statistically significant effect in the roughness of both acrylic resins tested. On the other hand, results showed that there was a statistically significant increase in the roughness of the samples of both types of acrylic resins after immersion in the cleaning agents. The long-term evaluation showed that the roughness of the samples after 1, 3 and 6 months of immersion in the cleaning agents was statistically significantly higher than that observed at baseline. This result was corroborated by SEM images, in which changes in the topography of the acrylic resins, mainly the degradation of the materials’ surface, was observed. This degradation could be attributed to the release of some products from within the polymeric material. The remaining methyl methacrylate in the polymerised material may be degraded when in aqueous solution by oxidation caused by oxygen and hydrolytic reactions,^14^ resulting in superficial changes. The results of this study are consistent with the results described by Cakan et al,^6^ who observed an increase in surface roughness of denture base and reline acrylic resin after soaking in disinfectant solutions for 8 h per day over a period of 140 days. According to the authors, the changes on the surface topography of the resins can be caused by components of disinfecting solutions, which can act as solvents or plasticisers. These findings also agree with other studies,^17^,^29^ in which changes in porosity and roughness of acrylic resins varied depending on the immersion time. In fact, according to Paranhos et al,^37^ increasing the exposure time to disinfection solutions can result in an increase in surface roughness. It is also important to mention that all solutions used in this study, except for distilled water, presented acid pH (Table 7). The pH affects degradation rates through catalysis.^16^ Thus, it is probable that the acid pH of the solutions also contributed to surface deterioration of the samples and, then, to the topographical changes, mainly on the samples immersed in apple vinegar, which had the lower pH values. This condition may have been enhanced by the storage time and daily exchange of the solutions.

Unexpectedly, other result observed was that after 1 month of immersion in the cleaning agents, the roughness of the samples was similar to that observed after 6 months of daily disinfection, being both statistically significantly higher than that observed after 3 months of immersion in the cleaning agents. These results could be explained by the use of independent samples, although the roughness was previously standardised.

The results have highlighted that surface changes also occurred in the samples immersed in distilled water. These results are in accordance with Cakan et al^6^ and Tuna et al,^46^ who reported that the absorption of water by acrylic resins can increase their surface roughness. Structural polymers are susceptible to damage in the form of cracks. Once cracks are formed within polymeric materials, the integrity of the structure can be statistically significantly compromised.^50^ Consequently, surface changes could promote the adhesion and subsequent biofilm formation.^53^

When the roughness of both types of acrylic resins was compared after chemical disinfection, the results showed that the mean values of roughness of the acrylic resin specimen were higher (4.09 μm) than those from the denture base acrylic resin specimen (4.02 μm). Despite being statistically significant, the difference (0.03 µm) may not be clinically relevant. This fact can be verified by the surface topography images (SEM). These findings are in accordance with Machado et al,^30^ who verified that the surface roughness of the denture base and reline acrylic resins was similar. These could be explained by the type of polymerisation of the materials, since the heat-polymerised materials have high conversion rate of monomer in polymer and low residual monomer content, while the reliner materials (Tokuyama Rebase) contains the monomer acetoacetoxyethyl methacrylate, which has high reactivity.^48^ These features reduce the amount of residual monomer released, resulting in surfaces less propitious to deformities. Furthermore, these results may be related to the fact that the samples of both resins were processed between two glass plates with standard roughness (3 µm), as recommended by Radford et al,^41^ in order to simulate the inner surface of the dentures.

Besides the evaluation of the topography of the acrylic resins, this study verified the influence of the surface changes on *C. albicans* biofilm formation in terms of cell density (CFU/ml values) and metabolism (Alamar Blue). The results showed that, for all groups, there was biofilm formation on the surface of the denture base and reline acrylic resins samples. Huh et al^18^ also found that the capacity of biofilm formation on acrylic resins samples was not influenced by daily use of denture cleansers. Despite superficial changes being found in the acrylic resins, this had no statistically significant effect on the CFU/ml values of the biofilms, regardless of the period of time and cleaning solution. Another investigation tested an in vitro method to simulate the dentures cleaning conditions for 1 year and also verified that their protocol (toothbrushing plus immersion in chemical solutions) did not affect the quantification of *C. albicans* cells within the biofilm.^27^ In the same way, in the Nikawa et al^33^ study, samples of denture base and reline acrylic resins were immersed in disinfecting solutions for 8 h per day during 180 days and the results showed no differences in biofilm formation by *C. albicans*. These results are further reinforced by the recent discovery that changes in roughness had no statistically significant effects on the adherence and biofilm cells of *C. albicans*.^10^ The precise mechanisms involved in the adherence of microorganisms to dentures are not totally recognised. Some authors^26^,^31^,^40^ highlighted the influence of hydrophobic interactions and electrostatic interactions that could influence the adherence of microorganisms to polymers.

The Alamar Blue assay measures the metabolic activity of the cells through mitochondrial enzymes and is a complementary test to the CFU/ml method, which evaluates the proliferative capacity of cells, regardless of their metabolic state. Despite the fact that the long-term disinfection with different cleaning solutions had no statistically significant effect on the cell density of the biofilms, the results showed that the metabolism of the cells in the biofilms was affected over time. At baseline, all experimental groups showed the highest fluorescence values in the Alamar Blue assay (Table 6), indicating high cellular metabolism. In fact, at that time, the samples were not immersed in any disinfection solutions, which could explain the higher metabolic activity of the cells. Nevertheless, as can be seen on our results, the fluorescence values obtained after 1, 3 and 6 months of chemical disinfection were statistically significantly lower than the values at baseline for the biofilms of all experimental groups. Thus, while the long-term disinfection with all cleaning solutions did not change the cell density of the biofilms, it decreased the metabolic activity of these cells. The metabolic activity of the cells is a direct indicator of their activity, so that the higher the metabolism of a biofilm, the higher the activity of the cells within this structure. When the microbial cells living in a biofilm are exposed to any kind of stress, such as a chemical solution, the dynamics of the biofilm is markedly changed. This has been linked to one of the major problems related to the presence of the biofilms, which is the resistance to antimicrobials. In this context, it has been shown that the reduced metabolic activity of a biofilm may be a possible mechanism of resistance to antimicrobial activity.^9^,^45^,^49^

The limitations of the present investigation include that the denture biofilm is complex microbial communities embedded in a polymeric matrix, and does not contain only *C. albicans* as tested in the methodology. In addition, the patients use brushing as the main method of cleaning their dentures in association with the chemical disinfection. However, there are no studies with the same periods of immersion and the same disinfectant solutions in the literature, thus the results described herein may be considered relevant. Furthermore, the resins’ wear mechanism is complex and long-term clinical observations should be made to accompaniment the results obtained in this study.

## Conclusion

Within the limitations of this study, it can be concluded that the roughness of both acrylic resins was affected by the disinfection in all cleaning agents. The periods of time had statistically significant effects on the roughness, which increased over time. In addition, the exposure to all solutions for 1, 3 and 6 months resulted in a decrease in metabolic activity of the cells within the biofilms.
